# Characterization of Chlorinated Aliphatic Hydrocarbons and Environmental Variables in a Shallow Groundwater in Shanghai Using Kriging Interpolation and Multifactorial Analysis

**DOI:** 10.1371/journal.pone.0142241

**Published:** 2015-11-13

**Authors:** Qiang Lu, Qi Shi Luo, Hui Li, Yong Di Liu, Ji Dong Gu, Kuang Fei Lin

**Affiliations:** 1 State Environmental Protection Key Laboratory of Environmental Risk Assessment and Control on Chemical Process, School of Resource and Environmental Engineering, East China University of Science and Technology, Shanghai 200237, P.R. China; 2 Shanghai Engineering Research Center of Contaminated Sites Remediation, Shanghai Institute for Design & Research on Environmental Engineering, Shanghai 200237, P.R. China; 3 School of Biological Sciences, Swire Institute of Marine Science, The University of Hong Kong, Pokfulam Road, Hong Kong SAR, P.R. China; East China Normal University, CHINA

## Abstract

CAHs, as a cleaning solvent, widely contaminated shallow groundwater with the development of manufacturing in China's Yangtze River Delta. This study focused on the distribution of CAHs, and correlations between CAHs and environmental variables in a shallow groundwater in Shanghai, using kriging interpolation and multifactorial analysis. The results showed that the overall CAHs plume area (above DIV) was approximately 9,000 m^2^ and located in the 2–4 m underground, DNAPL was accumulated at an area of approximately 1,400 m^2^ and located in the 6-8m sandy silt layer on the top of the muddy silty clay. Heatmap of PPC for CAHs and environmental variables showed that the correlation between “Fe^2+^” and most CAHs such as “1,1,1-TCA”, “1,1-DCA”, “1,1-DCE” and “%TCA” were significantly positive (*p<0*.*001*), but “%CA” and/or “%VC” was not, and “Cl^-^” was significantly positive correlated with “1,1-DCA” and “1,1-DCE” (*p<0*.*001*). The PCA demonstrated that the relative proportions of CAHs in groundwater were mostly controlled by the sources and the natural attenuation. In conclusion, the combination of geographical and chemometrics was helpful to establishing an aerial perspective of CAHs and identifying reasons for the accumulation of toxic dechlorination intermediates, and could become a useful tool for characterizing contaminated sites in general.

## Introduction

With the development of manufacturing in China's Yangtze River Delta, processing enterprises have exposed a large number of enterprise-situ soil and groundwater contamination incidents during the process when industries layout changed and moved out, resulting in land transfer and re-circulation. The unconfined water aquifers of eastern China coastal areas buried shallow, and was vulnerable to pollution by chlorinated aliphatic hydrocarbons (CAHs) Dense Non-Aqueous Phase Liquid (DNAPL) organic solvent which was emitted by processing enterprises. CAHs were widely used in industrial solvents and cleaning agents, 1,1,1-Trichloroethane (1,1,1-TCA) in particular, was mainly used as building materials, cleaning products, and metal degreasing agents. Due to the fact that improper handling and disposal led to leaks and spills, 1,1,1-TCA is a ubiquitous groundwater contaminant that has been detected at numerous waste disposal sites and industrial facilities [1]. Under anaerobic groundwater conditions, 1,1,1-TCA are usually reduced to less chlorinated products including 1,1-dichloroethane (1,1-DCA), 1,1-dichloroethene (1,1-DCE), chloroethane (CA), and vinyl chloride (VC) [2], these products can be more stable in groundwater than TCA, which frequently existed simultaneously in the sites contaminated with 1,1,1-TCA (Table 1).

**Table 1:** Property/characteristic of CAHs.

Property/characteristic	VC	CA	1,1-DCE	1,1-DCA	1,1,1-TCA	Reference
CAS Number	75-01-4	75-00-3	75-35-4	75-34-3	71-55-6	[3]
Molecular Weight	62.50	64.51	96.94	98.96	133.40	[3]
Specific gravity ^a^	0.911	0.898	1.218	1.176	1.339	[3]
Solubility in Water ^b^	1100	5740	2420	5500	4400	Noted below
K_H_ ^c^	0.0278	0.0069	0.0229	0.0059	0.0041	[3]
Log K_OW_ ^d^	0.60	1.43	1.48	1.79	2.47	[3]
Superfund active sites	549	654	254	337	393	[4]

^a^ g/ml, 20°C.

^b^ mg/L, 20°C, values from[3] except VC is for 25°C and 1,1-DCE is from[5].

^c^ K_H_ = Henry’s Law Constants, 25°C, atm-m^3^/mol, except CA is for 20°C.

^d^ K_OW_ = octanol-water Partition coefficient, 25°C, (mol L^-1^octanol)/(mol L^-1^water).

In the United States, 1,1,1-TCA has been found as a contaminant in at least 393 of the 1,315 National Priorities List (NPL) sites designated by the U.S. Environmental Protection Agency (data from a search of the NPL database in September 2012) [4]. The Bitterfeld/Wolfen region in Germany was contaminated because of the activities of the previous chemical industries, with about 25 km^2^ of soil and estimated 200 million m^3^ of groundwater polluted [6]. Before suitable remediation measures for the polluted area were scheduled, plenty of investigations were operated, which demonstrated that the area was contaminated primarily with CAHs and benzene, toluene, ethylbenzene and xylene (BTEX), hexachlorocyclohexane (HCH), chlorobenzenes, dichlorodiphenyltrichloroethane (DDT) isomers and many other contaminants [7], [8], [9], [10], [11], and the concentrations of CAHs (1,1,1-TCA and trichloroethene) came to several hundreds of milligrams per liter, exceeding effluent standards by several orders of magnitude [12].

In this study, we investigated the contaminants contents of the CAHs 1,1,1-TCA, 1,1-DCA, 1,1-DCE, CA, VC and groundwater parameters such as pH, electrical conductivity, chloride, nitrate, nitrite, sulfate, ferrous, calcium and magnesium in the aquifer of a contaminated site in Shanghai, China. The main objects of this study are (1) to establish an aerial perspective of the concentrations of 5 CAHs (1,1,1-TCA, 1,1-DCA, 1,1-DCE, CA and VC) within five sampling zones in a shallow contaminated groundwater in Shanghai; (2) to identify natural and anthropogenic origins of these CAHs contaminants; (3) to determine the relationship between the CAHs and the groundwater quality parameters. Geostatistical method (kriging) is used to support the first objective, while multifactorial analysis (MFA) is used to support the second objective and the third objective.

Due to the heterogeneity of groundwater and often the nature of accidental pollution process, the concentration of the pollutants may be quite different over very short distances. That it is often difficult to get a specific pollution status or to develop a conceptual model of the pollution site. In addition, the small data sets and not exhaustive samplings in the horizontal and vertical dimension were also the inevitable problems. Eliminating uncertainty is not feasible or cost-effective to estimate the risk and select the appropriate remedial methods. Thus, for the sake of quantification and reducing uncertainty, and minimizing the investigation costs, the use of geographical and chemometrics methods seems to be effective and reliable [13, 14]. To date, Kriging and multifactorial analysis (MFA) are two common examples of geostatistical and multivariate statistical methods, respectively.

Kriging uses the principle of spatial autocorrelation to interpolate point samples to areal maps [13, 14]. Given the field discretized by a grid, the value of a variable at each grid node is estimated by linear combination of the variable measured values, where the weight of each measured value depends on the distance from the node, according to the variogram function. The method has been widely employed in many environmental contamination studies [14, 15]. Multifactorial Analysis were integrated with geostatistic techniques. For instance, pearson pairwise correlation (PPC) and principal component analysis (PCA) are mathematical procedures that is frequently applied to environmental data, where datasets may be large and difficult to interpret. PCA helps visualise the complex inter-relationships between variables, identify the underlying patterns among a large number of contaminants, and based on these patterns, identify their origin [16, 17].

Together, the results from this study provide a comprehensive geospatial assessment of the 5 CAHs, correlations between CAHs and groundwater parameters in shallow groundwater in a contaminant site in Shanghai. It should be emphasized that this study was the first of its kind to investigate groundwater CAHs and parameters in such a contaminated manufacturing site in China's Yangtze River Delta. Results may help devise spatially informed guidelines for remediation efforts.

## Materials and Methods

### Site description

**Fig 1:**
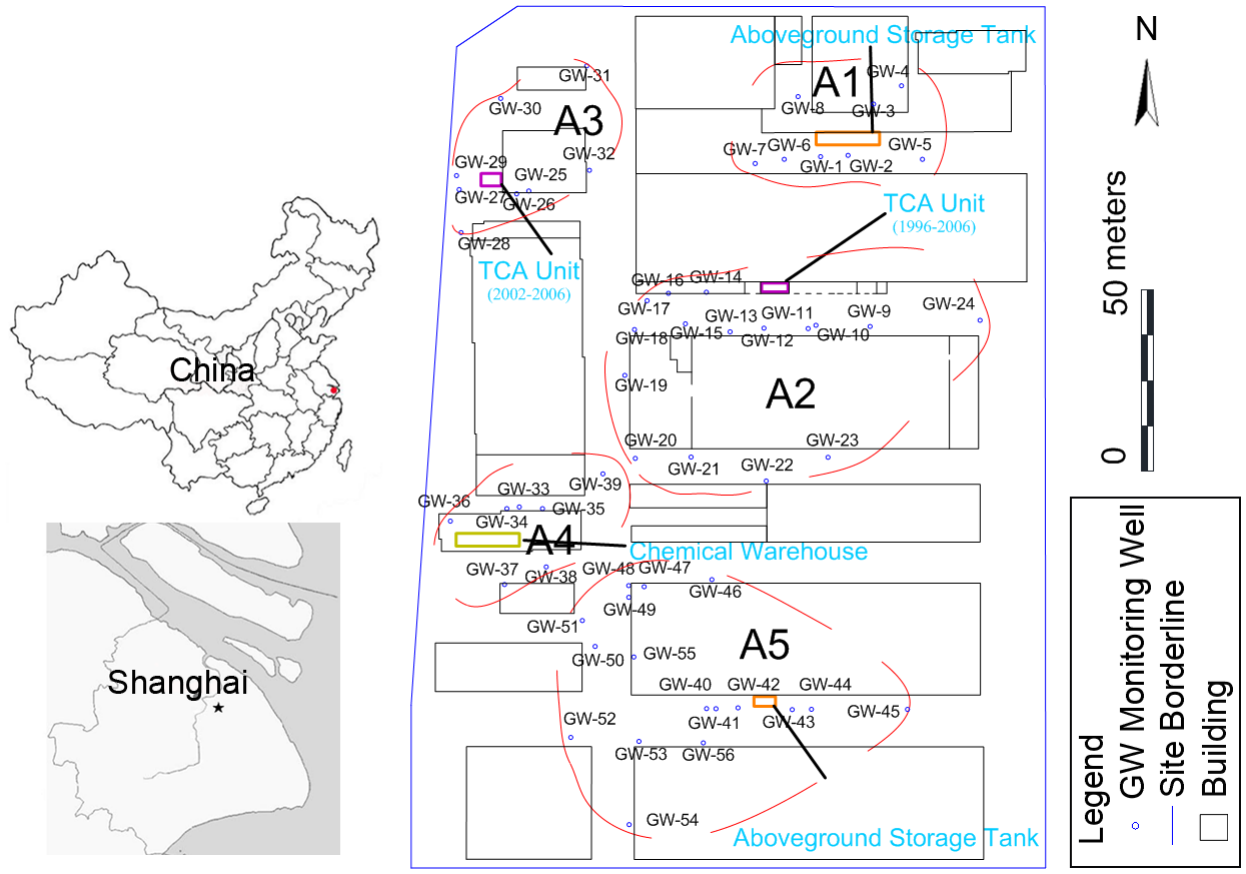
Map of the location of the investigated site.

The investigated site (The authors state clearly that no specific permissions were required for these locations because this site is a normal manufacturing factory without protected wildlife and protected area of land or sea. The authors confirm that the field studies did not involve endangered or protected species.) is located in Shanghai in East China and covers an irregular area of approximately 98,600 m^2^ and is mainly involved in manufacturing of car air conditioners since initial development in 1989 (Fig 1). During the production process, 1,1,1-TCA was used for degreasing metal parts until approximate 2008.

### Selection of wells

The previously collected information indicated that 1,1,1-TCA may generate 5 contaminated areas (A1, A2, A3, A4, A5) in the site. Therefore, in order to guarantee purposeful and accurate investigation, monitoring wells were installed in these 5 areas. As a result, 56 monitoring wells were installed for sampling the groundwater. Forty-Eight of 56 wells were screened at 2–4 m, eight of 56 wells were screened at deeper position as DNAPL wells, and 13 wells were chosen for laboratory tests of hydrogeological properties. The relative locations of the wells are described in Fig 1.

### Sampling of groundwater

The groundwater samples were obtained by using Sample ProTM (Type MP-SPK-6P-T, QED, USA) with as little agitation as possible (*Shanghai Engineering Research Center of Contaminated Sites Remediation*). To guarantee representative sampling, multiple (generally 3–5 times) volumes of the groundwater in the well before collection were pumped when the temperature (T), pH, dissolved oxygen concentration (DO), electrical conductivity (EC) had all stabilized. After pumping, the samples from each well were dispensed into 40-ml volatile organic analysis (VOA) vials for CAHs concentration analysis, and 20 ml high-density polyethylene bottles for groundwater quality parameters analysis. Groundwater samples were sealed with caps on PTFE-faced septa. Bubbles or headspace didn’t exist in the vials after they were capped and maintained on ice, then directly transported to the laboratories for chemical analysis.

### Reagents and standard materials

The 9 mmol/L Na_2_CO_3_ solution was selected as eluent for ion chromatography, and the reagents for DR/820 colorimeter were supplied by HACH USA. All solvents used throughout the analytical and instrumental phases for GC were pesticide grade. CAHs mixture standard solution was purchased from Accustandards (New Haven, CT, USA). Toluene-d_8_, 4-bromofluorobenzene and dibromofluoromethane as surrogate standards were also purchased from Accustandards.

### Chemical analysis

T, pH, DO and EC were monitored by Sample ProTM (Type MP-SPK-6P-T, QED, USA) during pumping. Chloridion (Cl^-^) concentration was determined by ion chromatography (Dionex ICS-1000, USA). Nitrite (NO_2_^-^), nitrate (NO_3_^-^), sulfate (SO_4_^2-^), ferrous (Fe^2+^), calcium (Ca^2+^), magnesium (Mg^2+^) were measured using DR/820 Colorimeter (HACH, USA). CAHs were analyzed by GC (Agilent 7890A) equipped with a split/splitless injector used in splitless mode (240°C), an electron capture detector (ECD) and a 60 m× 0.25 mm× 1.4 μm DB-VRX capillary column (J&W Scientific). The oven temperature program used was as follows: 0 min at 45°C, 12°C/min to 190°C and hold for 2 min. Nitrogen (ultra-pure) was used as a carrier gas and held at a constant flow rate of 2 ml/min and the ECD was operated at 260°C. The analysis was automated using Purge&Trap (Tekma Atomx) with a 5 ml purge tube. The operation conditions were as follows: purge temperature at 40°C, purge flow at 40 mL/min, purge time for 11 min, desorb temperature at 250°C and desorb time for 2 min.

### Quality assurance/quality control

All samples for CAHs analysis from different wells were spiked with surrogate standards of toluene-d8, 4-bromofluorobenzene and dibromofluoromethane. The recoveries for toluene-d_8_ ranged from 85.5% to 112.3% with an average of 98.2%, for 4-bromofluorobenzene ranged from 83.9% to 110.2% with an average of 95.6% and for dibromofluoromethane ranged from 88.1% to 115.4% with an average of 99.5%. The limit of detection (LOD) was defined as having a signal-to-noise ratio greater than 3.0 and was equal to 5 μg/L for each target chlorinated hydrocarbon. A method blank, a duplicate, and a laboratory control samples (LCS) were in each analytical batch. Differences between duplicate samples were typically less than 10%.

### Data analysis and geostatistial analysis

Prior to analysis, Samples below the LOD were assigned a value equal to the half of LOD [18].

Kriging interpolation was described by using surfer 11.0 (Golden Software Inc, 2012). MFA were carried out with R and PASW Statistics 18.0 (IBM SPSS Software Inc., USA).

## Results and Discussion

### Geology and hydrogeology at the site

**Fig 2:**
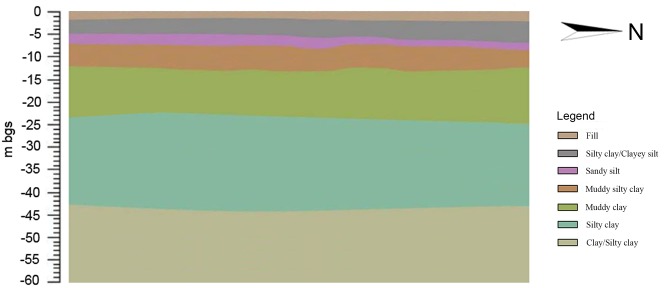
The Geology of the investigated site.

The stratum of the study area consisted of fill materials, clayey silt, sandy silt, muddy silty clay, muddy clay, silty clay and sandy silt (Fig 2). Fill materials occurred from the surface to depths ranging between 0.7–2.6 m and consisted of brown silty clay with occasional gravel, brick fragment. Clayey silt, ranged 1.1 m and 4.4 m in the thickness, was mainly brown, moist to saturated, and medium to low plasticity. Sandy silt, was grey, saturated, soft and low plasticity, ranging between 0.8 m and 4.0 m in thickness, and this layer was not continuous across the whole site as both conducting cone penetration test (CPT) and field observation indicated the lack of sandy silt layers in some locations. Muddy silty clay, ranged 2.5 m and 4.0 m in thickness, was mainly grey, saturated soft and low plasticity. Muddy clay was mainly grey, saturated, soft and low plasticity with very thin sand layer at some locations, and the top surface of muddy clay was encountered between 9.0 m and 10.2 m, while the bottom surface was between 18.4 m and 19.5 m. Silty clay was encountered at depth between 18.4 m and 19.5 m, while the bottom surface between 44.7 m and 46.8 m. Sandy silt was encountered at 44.7 m to 46.8 m extending to the CPT termination depth of 60.0 m.

As S1 Fig shows, the estimated groundwater flow direction at the site was generally from Southeast to Northwest in the shallow water bearing zone at the site, and the groundwater level generally located between 2.70 m and 3.35 m. One water bearing zone was encountered till muddy clay layers at the site.This water bearing zone was unconfined and was mainly recharged by the rain water and surface runoff.

Slug tests and laboratory tests results (Table 2) indicates that the horizontal hydraulic conductivities (K_H_) ranged from 2.15×10^−7^ cm/s (clayey layer) to 2.52×10^−4^ cm/s (clayey silt layer), while vertical hydraulic conductivities (K_V_) ranged from 1.25×10^−7^ cm/s (clayey layer) to 1.53×10^−4^ cm/s (silty layer). The hydraulic conductivities of the silty materials (clayey silt or sandy silt) are approximately one to two orders of magnitude greater than the underlain clayey materials (silty clay or clay). While the horizontal hydraulic conductivities are 1.2 to 4.5 times the vertical values in the same silty layer, the differences between the horizontal and vertical hydraulic conductivities are generally less in the clayey materials.

**Table 2:** The hydrogeology at the site.

Soil Type	Sampling Depth(m)	Particle Size		Physical Property					Plasticity/Liquid				Permeability(20°C)	
		Silt (5–74μm) /%	Clay (<5μm) /%	Water Content (W0) /%	Density (ρ0) /g·cm^-3^	Specific Weight (G)	Saturation (Sr) /%	Porosity Ration (e0)	Liquid Limit (W_L_) /%	Plasticity Limit (W_P_) /%	Plasticity Index (I_P_)	Liquid Index (I_L_)	K_V_ /cm·s^-1^	K_H_ /cm·s^-1^
Brownish Yellow Clay	2.0–2.3	69	31	24.3	2.03	2.77	93	0.73	39.5	20.9	18.1	0.21	1.21E-07	2.15E-07
Grey Silty Clay	4.0–4.3	83	17	30.5	1.91	2.73	94	0.91	32.6	19.1	12.3	0.86	9.75E-06	1.21E-05
Grey Sandy Silt	5.0–5.3	91	9	31.7	1.88	2.69	94	0.95	27.9	19.3	8.5	1.33	3.34E-05	1.52E-04
Grey Clayey Silt	6.0–6.3	86	14	28.9	1.93	2.71	96	0.91	30.3	20.5	9.2	0.87	1.51E-04	2.52E-04
Grey Muddy Silty Clay	7.0–7.3	81	19	48.8	1.72	2.72	95	1.39	36.2	22.1	13.3	1.87	2.91E-06	4.22E-06
Grey Clay	8.0–8.3	69	31	43.2	1.81	2.73	95	1.24	42.7	22.9	20.6	0.98	3.07E-07	3.71E-07
Grey Muddy Silty Clay	8.1–8.4	70	30	38.9	1.87	2.72	98	1.19	38.8	21.3	16.1	1.12	2.31E-06	3.17E-06
Grey Muddy Silty Clay	9.0–9.3	81	19	50.3	1.78	2.72	97	1.42	36.3	20.1	163	1.76	2.71E-06	4.13E-06
Grey Muddy Clay	10.0–10.3	67	33	49.1	1.77	2.73	99	1.41	46.9	24.1	21.1	1.24	3.61E-07	5.12E-07
Grey Clay	13.0–13.3	69	31	46.8	1.76	2.73	99	1.36	44.3	23.3	20.8	1.22	3.75E-07	5.06E-07
Grey Muddy Clay	14.0–14.3	67	33	48.1	1.75	2.73	99	1.4	47.7	25.7	22.6	1.14	3.67E-07	4.79E-07

These results indicated that the clayey materials below the shallow groundwater zone impeded downward migration of contaminants into the deeper zone. The most substantial way of migration of the contaminants is horizontal movement in the clayey silt and sandy silt layer [19].

### The CAHs horizontal distribution in the contaminated groundwater

In the investigated groundwater, five CAHs were detected: 1,1,1-TCA, 1,1-DCA, 1,1-DCE, CA, and VC, The collected data indicated that CAHs has caused contamination of groundwater at certain parts of the site (S1 Table).

A1 included 8 wells (from GW-01 to GW-08) and all wells were screened at 2–4 m. On the whole, 1,1,1-TCA was detected at concentrations ranging from n.d. (not detected) to 21.40 μg/L, and all detected concentrations were below the Dutch Intervention Values (DIV) [20] of 300 μg/L. 1,1-DCA was detected at concentrations ranging from n.d. to 6,010 μg/L. One of the 8 detected concentrations were above the DIV of 900 μg/L. 1,1-DCE was detected at concentrations ranging from n.d. to 1,080 μg/L. Four of the 8 detected concentrations were above the DIV of 10 μg/L. CA was detected at concentrations ranging from n.d. to 8,690 μg/L. No Dutch reference standard for CA was available for comparison. VC was detected at concentrations ranging from n.d. to 126 μg/L. Five of the 8 detected concentrations were above the DIV of 5 μg/L.

A2 included 16 wells, wells (GW-14, GW-16, GW-17, GW-19, GW-20, GW-21, GW-22, GW-23) were screened at 2–4 m; DNAPL wells were screened this way GW-09 at 7–8 m, GW-10 at 5–6 m, GW-11 at 6–7 m, GW-12 at 6–7 m, GW-13 at 6–7 m, GW-15 at 7–8 m, GW-18 at 7–8 m, and GW-24 at 7–8 m. On the whole, except DNAPL wells, 1,1,1-TCA was detected at concentrations ranging from n.d. to 354,000 μg/L. Three of the 8 detected concentrations were above the DIV of 300 μg/L. 1,1-DCA was detected at concentrations ranging from n.d. to 293,000 μg/L. Three of the 8 detected concentrations were above the DIV of 900 μg/L. 1,1-DCE was detected at concentrations ranging from n.d. to 3,090 μg/L. Three of the 8 detected concentrations were above the DIV of 10 μg/L. CA was detected at concentrations ranging from n.d. to 50,300 μg/L. VC was detected at concentrations ranging from n.d. to 670 μg/L. Three of the 8 detected concentrations were above the DIV of 5 μg/L.

A3 included 8 wells (from GW-25 to GW-32) and all wells were screened at 2–4 m. On the whole, 1,1,1-TCA was detected at concentrations ranging from n.d. to 232,000 μg/L. Two of the 8 concentrations were above the DIV of 300 μg/L. 1,1-DCA was detected at concentrations ranging from n.d. to 76,200 μg/L. Three of the 8 detected concentrations were above the DIV of 900 μg/L. 1,1-DCE was detected at concentrations ranging from n.d. to 3,890 μg/L. Four of the 8 detected concentrations were above the DIV of 10 μg/L. CA was detected at concentrations ranging from n.d. to 3,590 μg/L. VC was detected at concentrations ranging from n.d. to 180 μg/L. Three of the 8 detected concentrations were above the DIV of 5 μg/L.

A4 included 7 wells (from GW-33 to GW-39) and all wells were screened at 2–4 m. On the whole, 1,1,1-TCA was detected at concentrations ranging from n.d. to 795 μg/L. All detected concentrations were below the DIV of 300 μg/L. 1,1-DCA was detected at concentrations ranging from n.d. to 152,000 μg/L. Two of the 8 detected concentrations were above the DIV of 900 μg/L. 1,1-DCE was detected at concentrations ranging from n.d. to 257 μg/L. Three of the 8 detected concentrations were above the DIV of 10 μg/L. CA was detected at concentrations ranging from n.d. to 2,200 μg/L. VC was detected at concentrations ranging from n.d. to 42 μg/L. Three of the 8 detected concentrations were above the DIV of 5 μg/L.

A5 included 17 wells (from GW-40 to GW-56) and all wells were screened at 2–4 m. On the whole, 1,1,1-TCA was detected at concentrations ranging from n.d. to 34,700 μg/L. Four of the 17 concentrations were above the DIV of 300 μg/L. 1,1-DCA was detected at concentrations ranging from n.d. to 32,500 μg/L. Four of the 17 detected concentrations were above the DIV of 900 μg/L. 1,1-DCE was detected at concentrations ranging from n.d. to 12,200 μg/L. Eight of the 17 detected concentrations were above the DIV of 10 μg/L. CA was detected at concentrations ranging from n.d. to 33,700 μg/L. VC was detected at concentrations ranging from ND n.d. to 455 μg/L. Five of the 17 detected concentrations were above the DIV of 5 μg/L.

**Fig 3:**
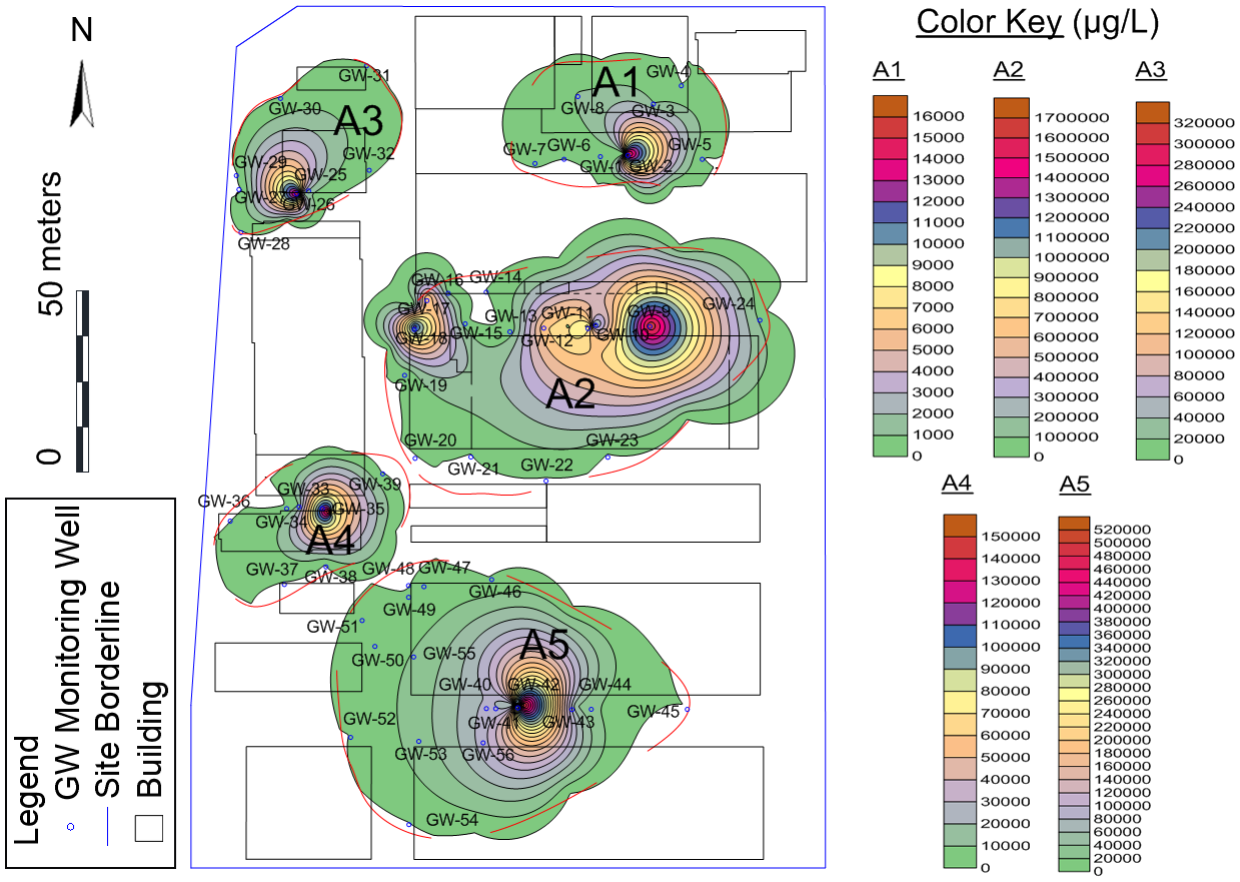
The horizontal distribution of CAHs in investigated site.

Kriging is applied to interpolate total CAHs concentrations of collected groundwater samples (screened at 2–4 m) in order to gain a horizontal distribution of the CAHs for A1, A2, A3, A4, A5 separately (Fig 3), CAHs delineation of five areas were completed. According to the contamination distribution and the operational history of the site, the potential on-site source areas for five concerned areas were identified. In A1, GW-02 showed the highest total concentration (15,869 μg/L) of 1,1,1-TCA (12 μg/L), 1,1-DCA (6,010 μg/L), 1,1-DCE (1,080 μg/L), CA (8,690 μg/L) and VC (77 μg/L), respectively exceeding the DIV. The location of GW-02 was used for chlorinated solvent drum storage in history, and the overall CAHs plume area (above DIV) was approximately 850 m^2^. In A2, except DNAPL wells, GW-17 showed the highest total concentration (701,060 μg/L) of 1,1,1-TCA (354,000 μg/L), 1,1-DCA (293,000 μg/L), 1,1-DCE (3,090 μg/L), CA (50,300 μg/L) and VC (670 μg/L), respectively exceeding the DIV. The well represented a TCA cleaning unit, which was operated during 1996 and 2006, and the overall CAHs plume area (above DIV) was approximately 3100 m^2^. In A3, GW-26 showed the highest total concentration (314,380 μg/L) of 1,1,1-TCA (232,000 μg/L), 1,1-DCA (76,200 μg/L), 1,1-DCE (3,890 μg/L), CA (2,110 μg/L) and VC (180 μg/L), respectively exceeding the DIV. The well also represented a TCA unit, which was operated during 2002 and 2006, and the overall CAHs plume area (above DIV) was approximately 800 m^2^. In A4, GW-35 showed the highest total concentration (154,886 μg/L) of 1,1,1-TCA (450 μg/L), 1,1-DCA (152,000 μg/L), 1,1-DCE (204 μg/L), CA (2,200 μg/L) and VC (32 μg/L), respectively exceeding the DIV. The well was used for historical Chemical Warehouse, and the overall CAHs plume area (above DIV) was approximately 760 m^2^. In A5, GW-41 showed the highest total concentration (91,245 μg/L) of 1,1,1-TCA (34,700 μg/L), 1,1-DCA (22,100 μg/L), 1,1-DCE (590 μg/L), CA (33,700 μg/L) and VC (155 μg/L), respectively exceeding the DIV. The location of the well was Outdoor Drum Storage containing TCA and/or oil/lubricants/diesel, and were historically stored on asphalt, paved or grassed areas, and the overall CAHs plume area (above DIV) was approximately 3,500 m^2^. Generally, the distribution of CAHs plume was spread from potential source to the surrounding, and the tendency of spread was obviously from the southeast to the northwest, and this fact may be due to its permeability and/or groundwater flows [21, 22].

### The CAHs DNAPL vertical distribution in the Area 2 contaminated groundwater

According to the one percent “rule of thumb”, the presence of DNAPL should be suspected if aqueous concentrations exceed 1% of a chemical's solubility in water [21, 23]. The CAHs solubility in water is presented in Table 1.

CAHs DNAPL was observed in the shallow groundwater zone at Area 2 (Old TCA Unit) during previous investigations. The objective of this DNAPL investigation was to assess the extent of CAHs DNAPL pool.

**Fig 4:**
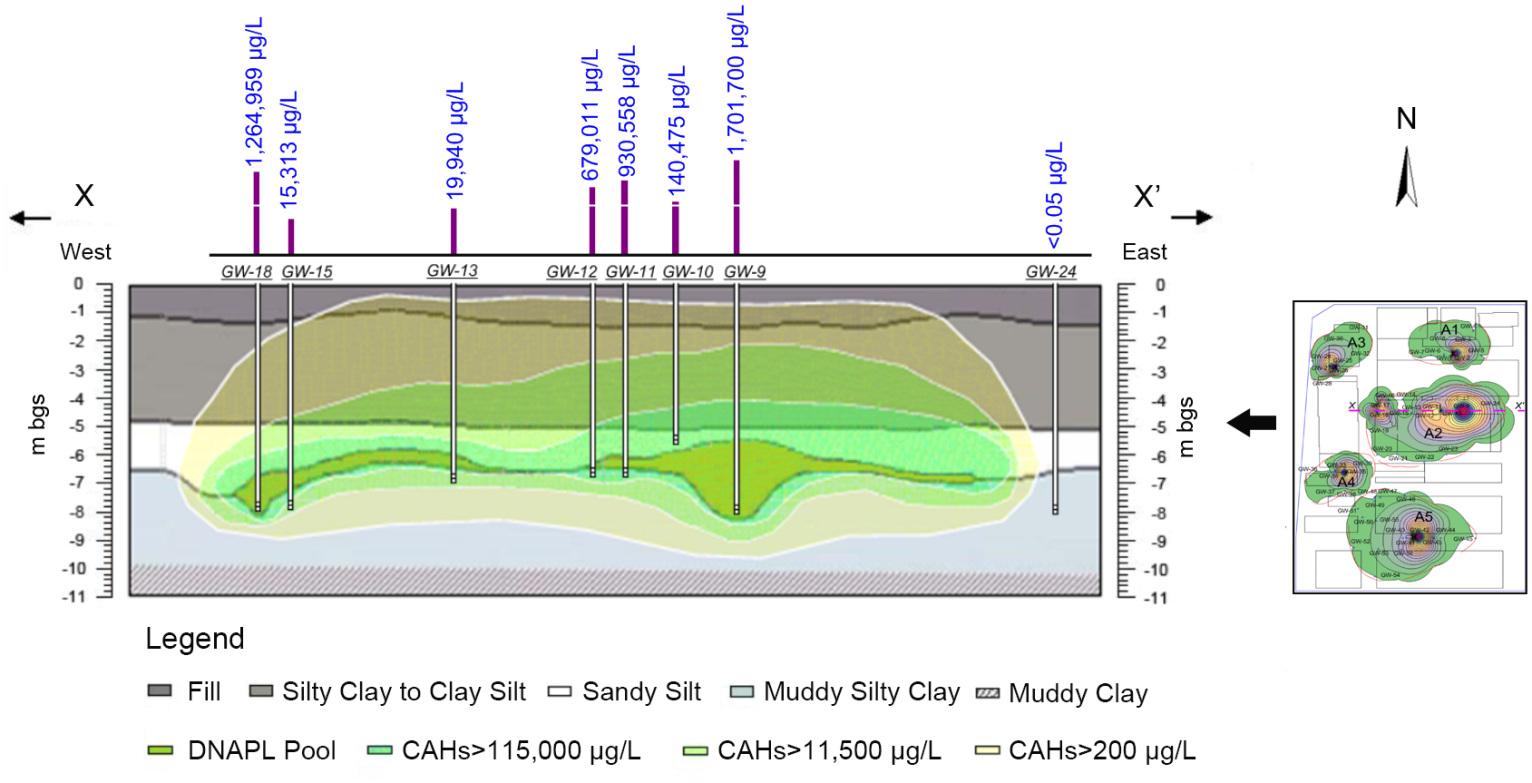
The DNAPL vertical distribution in the A2 area.

The DNAPL, located approximately 6–8 m (Fig 4), appeared to be present in the sandy silt layer on top of the muddy silty clay horizon. The data indicated that the thickest accumulations of DNAPL were found in wells locations where the bottom of the silty sand layer had lower elevation [24, 25].

The source area (DNAPL pool) was approximately 80 m long in east-west direction and approximately 24 m widest in south-north direction, with an area of approximately 1,400 m^2^. The overall CAHs plume area at Area 2 (above DIV) was approximately 4,500 m^2^. Nevertheless, DNAPL contributed to the continued presence of dissolved phase CAHs in groundwater [26].

The results of the DNAPL investigation supported a conceptual model for CAHs DNAPL distribution based on the hypothesis that a TCA release occurred at or near the former old TCA cleaning unit area. There may exist several locations where DNAPL historically may have entered the subsurface that contributes to considerable spatial variability of the source zone.

### Correlations between CAHs and Environmental Variables

**Fig 5:**
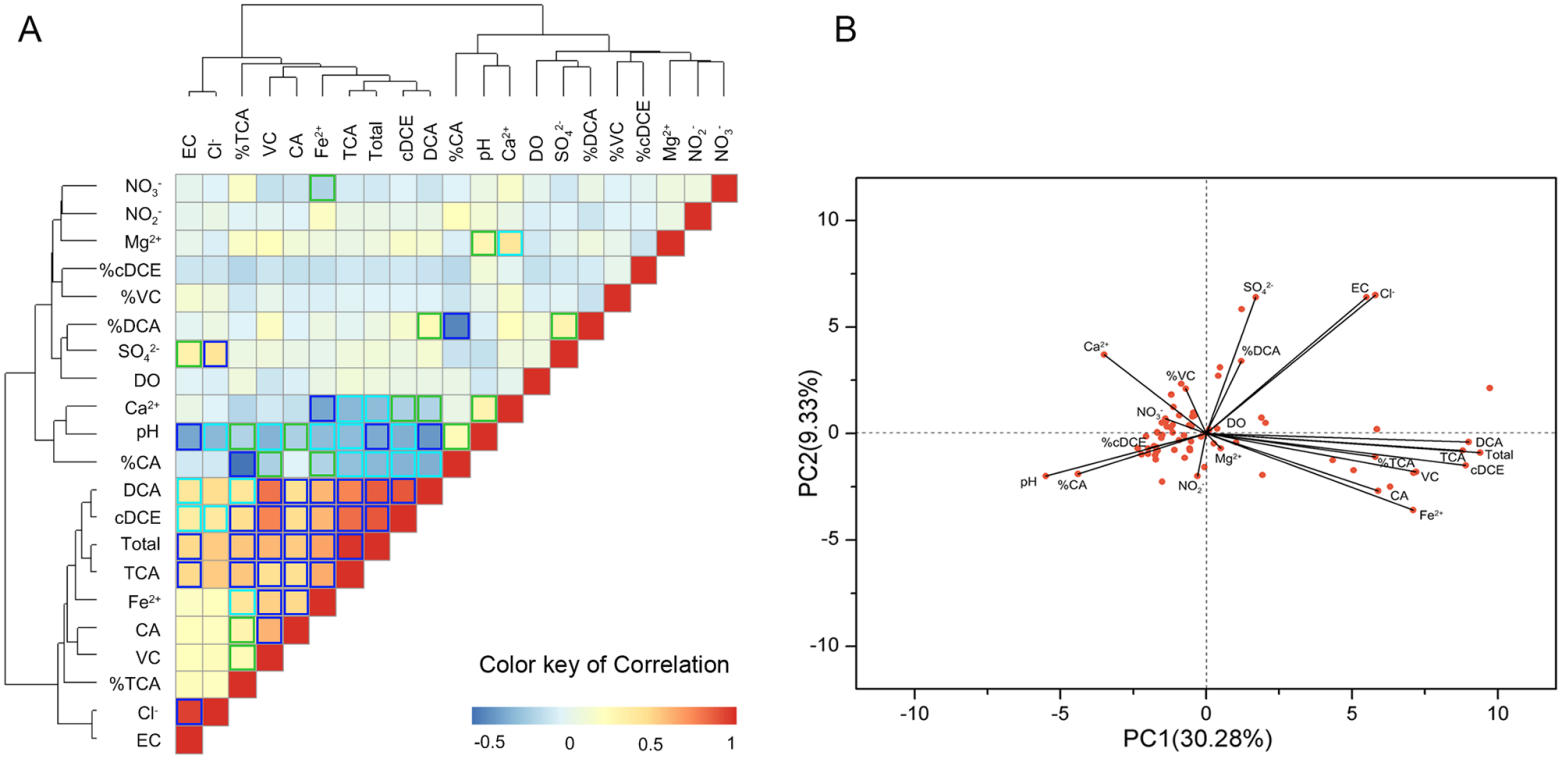
Heatmap of pairwise correlations and PCA between CAHs and environmental factors. Statistically significant correlations are indicated with a color surrounding the squares. Dark blue: p < 0.001; light blue: p < 0.01; green: p < 0.05.

The PPC was calculated to indicate the relationships between CAHs and environmental variables (Fig 5A, S2 Table). The correlations between “DO” and other variables were not significant, but overall, were following an almost weak negative pattern. The oxidation-reduction state analysis indicated that the main part of the contamination plume was under Fe(III)-reducing conditions[27]. Due to the dechlorination of high-chlorinated substance such as 1,1,1-TCA, 1,1-DCA and 1,1-DCE in the Fe(III)-reducing condition, “%CA” and/or “%VC” should have been accumulated at a specific sampling well and positively correlated with “Fe^2+^” [28, 29]. Nevertheless, “Fe^2+^” was significantly positive correlated with most CAHs such as “1,1,1-TCA”, “1,1-DCA”, “1,1-DCE” and with “%TCA” (*p<0*.*001*), but without “%CA” and/or “%VC”. This may resulted from the higher concentration of 1,1,1-TCA, 1,1-DCA and 1,1-DCE in most sampling wells, which usually presented a magnitude higher than CA and VC. What confirmed this was that “Cl^-^” was significant positively correlated with “1,1-DCA” and “1,1-DCE” (*p<0*.*001*), Cl^-^ is always the indicator for the dechlorination of CAHs [30].

The PCA was carried out by diagonalization of the correlation matrix, since all variables were scaled to variance unit and contributed equally. The problem of different numerical ranges of the original variables was avoided. In this study, two independent factors were extracted, explaining only 39.61% of the total variance (Fig 5B). The first one was responsible for 30.28% of the total variance and was perhaps mainly represented by the source of CAHs in the groundwater. Fig 5B showed that 1,1,1-TCA, 1,1-DCA, 1,1-DCE presented high positive loadings on the PC1, and the source of CAHs could cause high concentration of these three CAHs. PC2 explained 9.33% of the total variance and was perhaps mainly represented by the natural attenuation of CAHs in the groundwater, according to 1,1,1-TCA, 1,1-DCA, 1,1-DCE presenting negative loadings, while Cl^-^ presented high positive loadings on the PC2, and the natural attenuation of CAHs could decrease 1,1,1-TCA, 1,1-DCA and 1,1-DCE [29], as well as increase Cl^-^ [30].

## Conclusion

In this study, the CAHs (1,1,1-TCA, 1,1-DCA, 1,1-DCE, CA, VC) and groundwater parameters such as pH, electrical conductivity, chloride, nitrate, nitrite, sulfate, ferrous, calcium and magnesium in the groundwater of a contaminated site were investigated. Kriging was used to establish an aerial perspective of the concentrations of 5 CAHs (1,1,1-TCA, 1,1-DCA, 1,1-DCE, CA and VC) within five sampling zones. Expounded the horizontal distribution of the CAHs plume and the vertical distribution of DNAPL, combined with the hydrogeology of site, identified natural and anthropogenic origins of these CAHs contaminants.

The PPC analysis investigated the relationships between CAHs and environmental variables, and the results demonstrated that most groundwater samples were under Fe(III)-reducing conditions, the correlation between “Fe^2+^” and most CAHs such as “1,1,1-TCA”, “1,1-DCA”, “1,1-DCE” and “%TCA” were significant positive, but “%CA” and/or “%VC” was not, and “Cl^-^” was significantly positive correlated with “1,1-DCA” and “1,1-DCE”, which may speculate that it existed the natural attenuation of CAHs in the groundwater in this site, but the attenuation degree is low.

The results of the PCA allowed the reduction of the original data matrix to two important PCs explaining 39.61% of the total variance. 1,1,1-TCA, 1,1-DCA and 1,1-DCE with greatest positive loadings typically occurred as the source of CAHs in the groundwater, while 1,1,1-TCA, 1,1-DCA and 1,1-DCE with negative loadings, Cl^-^ with greatest positive loadings were generally more effected within the natural attenuation of CAHs in the groundwater. It demonstrated that the relative proportions of CAHs in groundwater were mostly controlled by the sources and the natural attenuation.

Results obtained in this work are intended to be an initial contribution for a more exhaustive investigation about geographical distribution of contamination patterns and their correlation with environmental variables in the shallow groundwater in Shanghai. This study also demonstrated the usefulness of the multifactorial analysis in hydrochemical studies.

## Supporting Information

S1 Fig The Groundwater elevation contour in the investigated site. (TIF)

S1 Table The concentration of CAHs and the environmental parameters. (DOC)

S2 Table Spearman’s correlation coefficient between CAHs and Environmental variables in the groundwater. (DOC)
