# Correction: Characterization of Chlorinated Aliphatic Hydrocarbons and Environmental Variables in a Shallow Groundwater in Shanghai Using Kriging Interpolation and Multifactorial Analysis

**DOI:** 10.1371/journal.pone.0144903

**Published:** 2015-12-16

**Authors:** Qiang Lu, Qi Shi Luo, Hui Li, Yong Di Liu, Ji Dong Gu, Kuang Fei Lin

The sixth author’s name is spelled incorrectly in the citation for the published article. The correct name is: Kuang Fei Lin. The correct citation is: Lu Q, Luo QS, Li H, Liu YD, Gu JD, Lin KF (2015) Characterization of Chlorinated Aliphatic Hydrocarbons and Environmental Variables in a Shallow Groundwater in Shanghai Using Kriging Interpolation and Multifactorial Analysis. PLoS ONE 10(11): e0142241. doi:10.1371/journal.pone.0142241

## References

[1] Lu Q, Luo QS, Li H, Liu YD, Gu JD, Fei Lin K (2015) Characterization of Chlorinated Aliphatic Hydrocarbons and Environmental Variables in a Shallow Groundwater in Shanghai Using Kriging Interpolation and Multifactorial Analysis. PLoS ONE 10(11): e0142241 doi: 10.1371/journal.pone.0142241 26565796 10.1371/journal.pone.0142241 PMC4643907

