# Supplementary figures and images for: Characterization of Chlorinated Aliphatic Hydrocarbons and Environmental Variables in a Shallow Groundwater in Shanghai Using Kriging Interpolation and Multifactorial Analysis
Source: PLoS One. 2015 Nov 13;10(11):e0142241. doi: 10.1371/journal.pone.0142241 (PMC4643907; doi:10.1371/journal.pone.0142241)

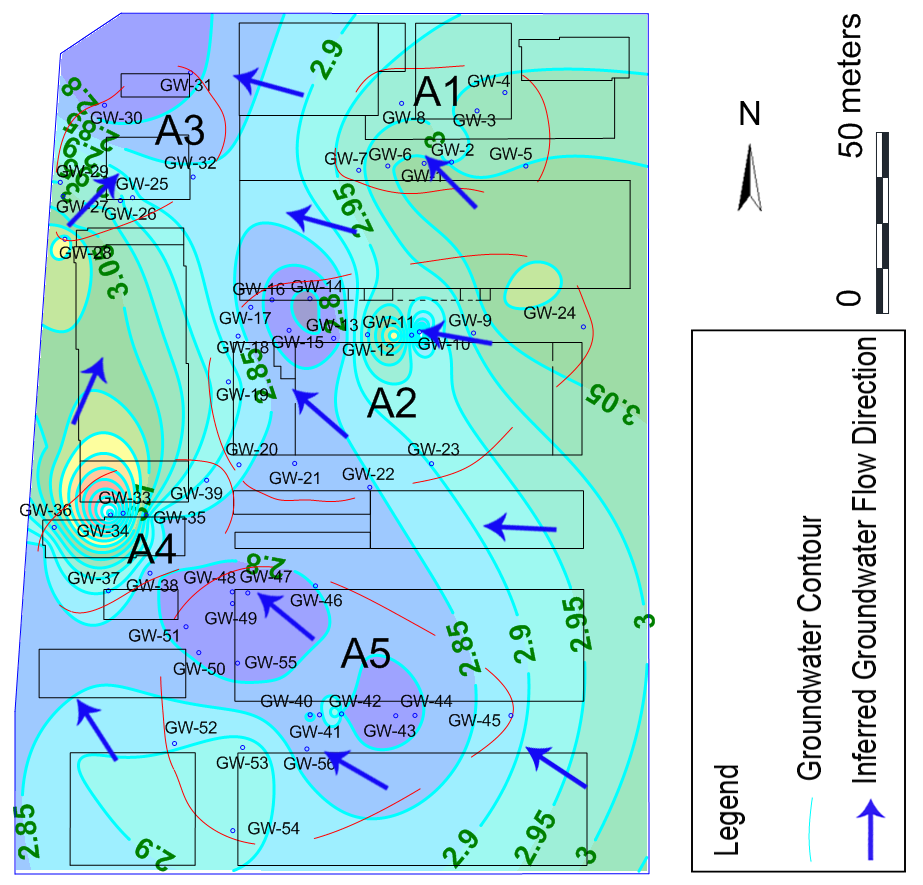

Supplement: S1 Fig — (TIF) [file pone.0142241.s001.tif]
