# Supplementary material for: Characterization of Chlorinated Aliphatic Hydrocarbons and Environmental Variables in a Shallow Groundwater in Shanghai Using Kriging Interpolation and Multifactorial Analysis
Source: PLoS One. 2015 Nov 13;10(11):e0142241. doi: 10.1371/journal.pone.0142241 (PMC4643907; doi:10.1371/journal.pone.0142241)
Supplement: S1 Table — (DOC) [file pone.0142241.s002.doc]

Table S1. The concentration of CAHs and the environmental parameters

| Well | VC | CA | 1,1-DCE | 1,1-DCA | 1,1,1-TCA | pH | DO | EC | Cl^-^ | NO_2_^-^ | NO_3_^-^ | SO_4_^2-^ | Fe^2+^ | Ca^2+^ | Mg^2+^ |
| --- | --- | --- | --- | --- | --- | --- | --- | --- | --- | --- | --- | --- | --- | --- | --- |
|  | μg·L^-1^ | μg·L^-1^ | μg·L^-1^ | μg·L^-1^ | μg·L^-1^ |  | mg·L^-1^ | μS·cm^-1^ | mg·L^-1^ | mg·L^-1^ | mg·L^-1^ | mg·L^-1^ | mg·L^-1^ | mg·L^-1^ | mg·L^-1^ |
| GW-1 | 15.50 | 405.00 | 12.20 | 141.00 | 21.40 | 7.9 | 0.15 | 530 | 110.36 | 0.001 | n.d. | 2.5 | 0.5 | 0.16 | 0.42 |
| GW-2 | 77.00 | 8690.00 | 1080.00 | 6010.00 | 12.00 | 7.6 | 0.23 | 650 | 132.67 | n.d. | n.d. | 4.5 | 30.2 | 0.23 | 0.41 |
| GW-3 | 126.00 | 384.00 | 393.00 | 23.50 | n.d. | 7.4 | 0.14 | 753 | 150.58 | n.d. | n.d. | 8.5 | 2.56 | 0.01 | 0.05 |
| GW-4 | n.d. | n.d. | n.d. | n.d. | 1.20 | 7.7 | 0.16 | 1668 | 755.86 | n.d. | n.d. | 0.2 | 0.5 | 0.24 | 0.26 |
| GW-5 | n.d. | n.d. | n.d. | 1.20 | 3.70 | 7.9 | 0.23 | 1643 | 599.28 | 0.001 | n.d. | 1.2 | 2.5 | 0.17 | 0.34 |
| GW-6 | n.d. | n.d. | 21.50 | 1.50 | n.d. | 7.5 | 0.14 | 479 | 139.93 | 0.001 | 0 | 1.2 | 0.5 | 0.12 | 0.13 |
| GW-7 | 107.00 | n.d. | n.d. | n.d. | n.d. | 7.5 | 0.15 | 3409 | 1607.77 | n.d. | n.d. | 5.2 | 3.1 | 0.31 | 0.24 |
| GW-8 | 14.30 | 1070.00 | 3.10 | 2.70 | 2.30 | 7.7 | 0.18 | 1039 | 313.59 | 0.001 | n.d. | 56.5 | 5.2 | 0.15 | 0.24 |
| GW-9 | 212.00 | 298.00 | 5190.00 | 326000.00 | 1370000.00 | 7.1 | 0.21 | 8413 | 6791.3 | 0.012 | n.d. | 35 | 45.35 | 0.01 | 0.21 |
| GW-10 | 154.00 | 7780.00 | 741.00 | 35600.00 | 96200.00 | 7.2 | 0.13 | 1212 | 192.31 | 0.012 | n.d. | 5 | 36.5 | 0.31 | 0.86 |
| GW-11 | 968.00 | 104000.00 | 5590.00 | 272000.00 | 548000.00 | 6.8 | 0.15 | 2985 | 1953.2 | 0.013 | n.d. | 40.5 | 30.4 | n.d. | n.d. |
| GW-12 | 321.00 | 130000.00 | 1690.00 | 107000.00 | 440000.00 | 7.1 | 0.23 | 2424 | 1250.9 | n.d. | n.d. | 36.5 | 32.1 | 0.01 | 0.03 |
| GW-13 | n.d. | 5560.00 | n.d. | 12500.00 | 1880.00 | 7.8 | 0.17 | 679 | 179.75 | 0.002 | n.d. | 6.5 | 32.2 | 0.12 | 0.41 |
| GW-14 | n.d. | 574.00 | n.d. | 1.80 | 1.40 | 7.9 | 0.16 | 3533 | 1890.71 | n.d. | n.d. | 85.2 | 5.25 | 0.35 | 0.81 |
| GW-15 | 93.00 | 3520.00 | 200.00 | 3500.00 | 8000.00 | 7.6 | 0.14 | 403 | 135.52 | 0.005 | n.d. | 85.4 | 16.51 | 0.02 | n.d. |
| GW-16 | n.d. | 43500.00 | 105.00 | 4460.00 | 2320.00 | 7.5 | 0.21 | 891 | 301.44 | 0.001 | n.d. | 23.1 | 42.3 | n.d. | n.d. |
| GW-17 | 670.00 | 50300.00 | 3090.00 | 293000.00 | 354000.00 | 6.7 | 0.13 | 3945 | 3022.58 | 0.015 | n.d. | 25.6 | 28.52 | n.d. | n.d. |
| GW-18 | 579.00 | 82700.00 | 4680.00 | 206000.00 | 971000.00 | 7.2 | 0.21 | 2712 | 1199.34 | 0.001 | n.d. | 25.5 | 42.35 | 0.03 | 0.02 |
| GW-19 | 6.58 | 1180.00 | 129.00 | 8280.00 | 922.00 | 7.7 | 0.14 | 845.6 | 265.3 | 0.001 | n.d. | 2.5 | 0.2 | 0.23 | 0.41 |
| GW-20 | n.d. | n.d. | n.d. | n.d. | 0.60 | 7.7 | 0.24 | 2526 | 990.96 | n.d. | n.d. | n.d. | 0.52 | 0.13 | 0.14 |
| GW-21 | n.d. | 9.10 | n.d. | 58.60 | 137.00 | 7.7 | 0.17 | 670 | 47.46 | 0.002 | 0.01 | 4.6 | 2.5 | 0.15 | 0.17 |
| GW-22 | n.d. | n.d. | n.d. | n.d. | n.d. | 7.6 | 0.19 | 3286 | 956.81 | 0.001 | n.d. | n.d. | 0.16 | 0.15 | 0.38 |
| GW-23 | n.d. | n.d. | n.d. | n.d. | n.d. | 7.6 | 0.15 | 3841 | 1204.41 | 0.003 | n.d. | n.d. | 0.21 | 0.13 | 0.42 |
| GW-24 | n.d. | n.d. | n.d. | n.d. | 5.60 | 7.6 | 0.17 | 2785 | 702.29 | 0.005 | 0.02 | n.d. | 0.06 | 0.24 | 0.89 |
| GW-25 | 47.30 | 3590.00 | 277.60 | 3220.00 | 1130.00 | 6.8 | 0.24 | 2341 | 526.3 | 0.004 | 0.01 | 12.5 | 8.52 | 0.21 | 0.16 |
| GW-26 | n.d. | 2110.00 | 3890.00 | 76200.00 | 232000.00 | 7.6 | 0.17 | 3265.8 | 1862.7 | 0.005 | 0.01 | 23.5 | 2.3 | 0.13 | 0.16 |
| GW-27 | n.d. | n.d. | n.d. | n.d. | n.d. | 7.7 | 0.19 | 10676 | 5324.74 | 0.012 | 0 | 0.8 | 0.6 | 0.21 | 0.15 |
| GW-28 | n.d. | n.d. | n.d. | n.d. | n.d. | 7.8 | 0.23 | 1046 | 201.95 | 0.002 | 0 | 0.6 | 3.2 | 0.12 | 0.41 |
| GW-29 | n.d. | n.d. | n.d. | n.d. | n.d. | 7.6 | 0.16 | 1520 | 312.57 | 0 | 0 | 0 | 2.5 | 0.32 | 0.21 |
| GW-30 | n.d. | n.d. | n.d. | n.d. | n.d. | 7.1 | 0.19 | 781 | 168.3 | 0.003 | n.d. | 21.5 | 5.25 | 0.14 | 0.32 |
| GW-31 | 2.30 | 72.90 | 16.10 | 2.20 | n.d. | 8 | 0.24 | 593 | 111.05 | n.d. | n.d. | 1.3 | 0.62 | 0.14 | 0.16 |
| GW-32 | 24.50 | 799.00 | 252.00 | 2030.00 | 105.00 | 7.7 | 0.18 | 695 | 137.06 | 0.001 | n.d. | 5.5 | 5.8 | 0.31 | 0.21 |
| GW-33 | 41.70 | 4330.00 | 256.60 | 3150.00 | 795.00 | 6.8 | 0.24 | 2341 | 526.3 | 0.004 | 0.01 | 12.5 | 8.52 | 0.21 | 0.16 |
| GW-34 | 9.14 | 567.00 | 12.91 | 4.13 | n.d. | 7.1 | 0.17 | 2341.5 | 526.3 | 0.006 | n.d. | 7.6 | 0.5 | 0.23 | 0.12 |
| GW-35 | 31.66 | 2200.00 | 204.00 | 152000.00 | n.d. | 7.2 | 0.23 | 2921.5 | 1563.5 | 0.001 | n.d. | 45.3 | 0.4 | 0.36 | 0.16 |
| GW-36 | n.d. | n.d. | n.d. | n.d. | n.d. | 7.9 | 0.14 | 698.3 | 125.5 | 0.006 | n.d. | 0.5 | 0.2 | 0.15 | 0.23 |
| GW-37 | n.d. | 3.77 | n.d. | 1.22 | 1.56 | 8 | 0.21 | 186.4 | 56.8 | 0.015 | 0 | 0 | 0.23 | 0.26 | 0.34 |
| GW-38 | n.d. | 195.20 | 0.56 | 1.96 | n.d. | 7.9 | 0.16 | 2563.4 | 654.5 | 0.004 | n.d. | 8.6 | 0.5 | 0.21 | 0.1 |
| GW-39 | n.d. | 5.00 | n.d. | 1.78 | 2.20 | 7.4 | 0.16 | 486.5 | 134.5 | 0.012 | n.d. | 0.1 | 0.01 | 0.16 | 0.15 |
| GW-40 | n.d. | 80.12 | n.d. | 0.81 | n.d. | 6.7 | 0.21 | 2856.4 | 1243.2 | 0.015 | 0 | 4.2 | 6.65 | 0.14 | 0.06 |
| GW-41 | 154.96 | 33700.00 | 590.00 | 22100.00 | 34700.00 | 7.5 | 0.16 | 2563.5 | 856.3 | 0.003 | n.d. | 5.6 | 1.5 | 0.21 | 0.34 |
| GW-42 | 455.00 | 25700.00 | 2270.00 | 32500.00 | 919.00 | 7.7 | 0.16 | 1124.5 | 456.5 | n.d. | n.d. | 0.3 | 12.5 | 0.25 | 0.14 |
| GW-43 | 16.00 | 30200.00 | 27.89 | 12200.00 | 557.00 | 7.6 | 0.18 | 1563.5 | 568.5 | n.d. | n.d. | 0.2 | 0.05 | 0.25 | 0.01 |
| GW-44 | n.d. | n.d. | 7.55 | 4.54 | 1.74 | 7.6 | 0.13 | 2986.5 | 1324.6 | 0.003 | n.d. | 5.3 | 8.56 | 0.21 | 0.08 |
| GW-45 | n.d. | 3.58 | n.d. | 0.74 | 1.03 | 7.8 | 0.13 | 134.7 | 45.6 | 0.013 | 0.02 | 0.5 | 0.64 | 0.24 | 0.02 |
| GW-46 | n.d. | 3.84 | n.d. | 1.04 | 1.38 | 7.6 | 0.15 | 658.5 | 156.3 | n.d. | n.d. | 0.2 | 0.41 | 0.12 | 0.16 |
| GW-47 | n.d. | 9.49 | 11.75 | 4.89 | 1.65 | 7.7 | 0.18 | 725.4 | 56.5 | 0.012 | 0.01 | 0.2 | 0.3 | 0.32 | 0.12 |
| GW-48 | 7.49 | 146.10 | 43.34 | 240.80 | 38.57 | 7.9 | 0.17 | 756.5 | 234.5 | 0.001 | 0 | 62 | 0 | 0.21 | 0.89 |
| GW-49 | 3.22 | 34.42 | 50.86 | 92.70 | 53.73 | 7.9 | 0.15 | 563.5 | 56.5 | n.d. | n.d. | 0.6 | 0.21 | 0.23 | 0.15 |
| GW-50 | n.d. | 9.47 | 2.83 | 261.40 | 218.50 | 7.4 | 0.23 | 721.4 | 53.5 | 0.012 | n.d. | 0.5 | 0.32 | 0.13 | 0.06 |
| GW-51 | n.d. | 3.37 | 3.12 | 1.67 | 1.03 | 7.7 | 0.21 | 156.8 | 38.7 | 0.002 | n.d. | n.d. | n.d. | 0.14 | 0.23 |
| GW-52 | n.d. | 3.71 | n.d. | 0.75 | 1.01 | 7.7 | 0.23 | 236.7 | 48.3 | n.d. | n.d. | 1.2 | 0 | 0.02 | 0.04 |
| GW-53 | n.d. | 3.02 | n.d. | 6.32 | 1.20 | 7.9 | 0.16 | 583.6 | 135.6 | 0.012 | 0.01 | 0.5 | 0.45 | 0.21 | 0.14 |
| GW-54 | n.d. | 3.62 | n.d. | 0.82 | 0.96 | 7.5 | 0.14 | 113.6 | 36.1 | 0.005 | n.d. | 0.8 | 0.42 | 0.12 | 0.04 |
| GW-55 | 32.57 | 168.10 | 45.14 | 14.18 | 5.25 | 7.9 | 0.24 | 658.5 | 168.5 | 0.003 | n.d. | 5.8 | 0.2 | 0.14 | 0.05 |
| GW-56 | n.d. | 24.77 | 37.25 | 1880.00 | 5000.00 | 7.7 | 0.23 | 786.5 | 256.8 | 0.003 | n.d. | 54 | 0.5 | 0.54 | 0.32 |

n.d.: not detected (below the LOD)
