# Supplementary material for: Characterization of Chlorinated Aliphatic Hydrocarbons and Environmental Variables in a Shallow Groundwater in Shanghai Using Kriging Interpolation and Multifactorial Analysis
Source: PLoS One. 2015 Nov 13;10(11):e0142241. doi: 10.1371/journal.pone.0142241 (PMC4643907; doi:10.1371/journal.pone.0142241)
Supplement: S2 Table — (DOC) [file pone.0142241.s003.doc]

Table S2. Spearman’s correlation coefficient between CAHs and Environmental variables in the groundwater

|  | pH | DO | EC | Cl^-^ | NO_2_^-^ | NO_3_^-^ | SO_4_^2-^ | Fe^2+^ | Ca^2+^ | Mg^2+^ | VC | CA | DCE | DCA | TCA | %VC | %CA | %DCE | %DCA | %TCA |
| --- | --- | --- | --- | --- | --- | --- | --- | --- | --- | --- | --- | --- | --- | --- | --- | --- | --- | --- | --- | --- |
| pH | 1.000 |  |  |  |  |  |  |  |  |  |  |  |  |  |  |  |  |  |  |  |
| DO | -0.126 | 1.000 |  |  |  |  |  |  |  |  |  |  |  |  |  |  |  |  |  |  |
| EC | -0.420^***^ | -0.063 | 1.000 |  |  |  |  |  |  |  |  |  |  |  |  |  |  |  |  |  |
| Cl^-^ | -0.357^**^ | -0.081 | 0.948^***^ | 1.000 |  |  |  |  |  |  |  |  |  |  |  |  |  |  |  |  |
| NO_2_^-^ | 0.082 | -0.081 | -0.026 | 0.010 | 1.000 |  |  |  |  |  |  |  |  |  |  |  |  |  |  |  |
| NO_3_^-^ | 0.020 | -0.020 | -0.022 | -0.070 | 0.043 | 1.000 |  |  |  |  |  |  |  |  |  |  |  |  |  |  |
| SO_4_^2-^ | -0.172 | 0.034 | 0.304^*^ | 0.408^***^ | -0.080 | -0.128 | 1.000 |  |  |  |  |  |  |  |  |  |  |  |  |  |
| Fe^2+^ | -0.343^**^ | 0.015 | 0.164 | 0.195 | 0.172 | -0.236^*^ | -0.001 | 1.000 |  |  |  |  |  |  |  |  |  |  |  |  |
| Ca^2+^ | 0.290^*^ | -0.017 | 0.002 | -0.080 | 0.012 | 0.141 | 0.055 | -0.423^***^ | 1.000 |  |  |  |  |  |  |  |  |  |  |  |
| Mg^2+^ | 0.269^*^ | -0.124 | -0.015 | -0.084 | 0.022 | 0.044 | -0.023 | 0.042 | 0.395^**^ | 1.000 |  |  |  |  |  |  |  |  |  |  |
| VC | -0.387^**^ | -0.143 | 0.196 | 0.210 | -0.045 | -0.167 | 0.059 | 0.502^***^ | -0.118 | 0.208 | 1.000 |  |  |  |  |  |  |  |  |  |
| CA | -0.255^*^ | -0.087 | 0.195 | 0.185 | -0.074 | -0.131 | 0.006 | 0.472^***^ | -0.165 | 0.065 | 0.607^***^ | 1.000 |  |  |  |  |  |  |  |  |
| DCE | -0.393^**^ | 0.004 | 0.343^**^ | 0.373^**^ | -0.007 | -0.071 | 0.027 | 0.592^***^ | -0.252^*^ | 0.129 | 0.746^***^ | 0.460^***^ | 1.000 |  |  |  |  |  |  |  |
| DCA | -0.481^***^ | 0.014 | 0.393^**^ | 0.452^***^ | 0.006 | -0.132 | 0.112 | 0.583^***^ | -0.233^*^ | 0.108 | 0.803^***^ | 0.437^***^ | 0.879^***^ | 1.000 |  |  |  |  |  |  |
| TCA | -0.346^**^ | 0.069 | 0.473^***^ | 0.525^***^ | 0.009 | -0.107 | 0.052 | 0.621^***^ | -0.358^**^ | 0.013 | 0.438^***^ | 0.439^***^ | 0.807^***^ | 0.754^***^ | 1.000 |  |  |  |  |  |
| %VC | -0.003 | -0.091 | 0.108 | 0.059 | -0.065 | -0.073 | -0.056 | -0.094 | 0.125 | -0.011 | -0.012 | -0.090 | -0.075 | -0.078 | -0.064 | 1.000 |  |  |  |  |
| %CA | 0.230^*^ | 0.001 | -0.117 | -0.124 | 0.198 | -0.078 | -0.150 | -0.228^*^ | 0.011 | -0.086 | -0.250^*^ | -0.077 | -0.372^**^ | -0.393^**^ | -0.331^**^ | -0.137 | 1.000 |  |  |  |
| %DCE | 0.072 | -0.165 | -0.144 | -0.139 | -0.078 | -0.038 | -0.129 | -0.172 | -0.048 | -0.139 | -0.131 | -0.177 | -0.126 | -0.148 | -0.121 | -0.005 | -0.198 | 1.000 |  |  |
| %DCA | -0.095 | 0.075 | -0.044 | 0.024 | -0.131 | 0.002 | 0.289^*^ | 0.023 | 0.202 | 0.037 | 0.174 | -0.066 | 0.118 | 0.237^*^ | -0.044 | -0.150 | -0.536^***^ | -0.100 | 1.000 |  |
| %TCA | -0.242^*^ | 0.064 | 0.216 | 0.185 | -0.063 | 0.148 | 0.031 | 0.397^**^ | -0.215 | 0.156 | 0.238^*^ | 0.288^*^ | 0.461^***^ | 0.398^**^ | 0.542^***^ | -0.123 | -0.613^***^ | -0.217 | -0.079 | 1.000 |

***. Correlation is significant at the 0.001 level (2-tailed).

**. Correlation is significant at the 0.01 level (2-tailed).

*. Correlation is significant at the 0.05 level (2-tailed).
